# Why Foot-and-Mouth Disease-Free with Vaccination Should Be Equivalent to Foot-and-Mouth Disease-Free without Vaccination

**DOI:** 10.3390/vetsci11060281

**Published:** 2024-06-19

**Authors:** Pedro Moura, Ulrich Kihm, Alejandro Schudel, Ingrid Bergmann, Patrik Buholzer

**Affiliations:** 1 SAFOSO AG, CH-3097 Liebefeld, Switzerland; 2 TAFS Forum, CH-3097 Liebefeld, Switzerland; 3 Fundación Prosaia, Buenos Aires C1061, Argentina; 4 Centro de Virología Humana y Animal (CEVHAN), Consejo Nacional de Investigaciones Científicas y Técnicas (CONICET), Universidad Abierta Interamericana (UAI), Buenos Aires C1287, Argentina

**Keywords:** foot-and-mouth disease, control, eradication, free sanitary status

## Abstract

**Simple Summary:**

Foot-and-Mouth Disease is one of the most relevant animal diseases and remains of global concern. The World Organization for Animal Health has specified two sanitary statuses that assure freedom from Foot-and-Mouth Disease: “Foot-and-Mouth Disease-free with vaccination” and “Foot-and-Mouth Disease-free without vaccination”. Over the recent decades, technological improvements in vaccines and diagnostics allow demonstration of freedom from virus circulation regardless of vaccination status. Therefore, the distinction between the two Foot-and-Mouth Disease-free statuses is outdated and no longer appropriate. The authors present their collective view on the position of recognizing both Foot-and-Mouth Disease-free statuses as equivalent in terms of sanitary protection guarantees. This will be made clear by highlighting how Foot-and-Mouth Disease freedom is achieved and how vaccines, diagnostic tools, and disease control capacity have improved, allowing reliable demonstration of freedom from Foot-and-Mouth Disease virus circulation. The prerequisites to recognizing the two statuses as equivalent, as well as disease control issues, will be discussed. They further illustrate that Foot-and-Mouth Disease-free status with vaccination can be considered Foot-and-Mouth Disease-free with active prevention. Recognizing the two Foot-and-Mouth Disease statuses as equivalent will allow countries to choose if they aim for freedom with or without vaccination, based on their national and regional epidemiological risk and without compromising the safety of the production system.

**Abstract:**

Foot-and-Mouth Disease (FMD) is still one of the most relevant animal diseases and remains of global concern. The World Organization for Animal Health (WOAH) has specified two sanitary statuses that assure freedom from FMD: a country or zone can be free from FMD either with or without vaccination. To obtain either of the two statuses, absence of virus circulation must be shown. The standards set by WOAH are used for trade negotiations. During recent decades, different tools and approaches were developed to control FMD, including vaccines, diagnostics, and the Progressive Control Pathway for FMD. These tools improved over time, and nowadays high-quality, reliable vaccines and specific diagnostics are available to efficiently control and detect the infection, even in vaccinated populations. Due to these improvements, it is no longer justifiable to treat the two FMD-free statuses differently. The distinction between the statuses provides wrong incentives and tempts countries to take increased risks by stopping vaccination to improve their trade conditions, which can have potentially devastating consequences. The decision to stop vaccination should only be made on the basis of a careful and comprehensive analysis of the local and regional epidemiological situation. This paper presents the perspective that member countries and WOAH should recognize the two FMD-free statuses as equivalent.

## 1. Introduction

Foot-and-Mouth Disease (FMD) remains a global concern. Although typically not fatal, it is one of the most economically feared animal diseases [1]. Today, about two thirds of countries are endemically affected by FMD, mainly in Asia, the Middle East, and Africa [2]. In addition to appropriate biosecurity and other disease control and prevention measures, vaccines are a key element, either to control or to prevent the disease.

The World Organisation for Animal Health (WOAH) has the mandate from the World Trade Organization (WTO) to officially recognize disease-free areas or countries for trade purposes. Therefore, the official recognition of animal health statuses is of great importance for international trade [3]. To achieve a certain status, countries need to submit documented evidence of strict compliance with the requirements of WOAH [4]. Specifically, a country applying for an official FMD status needs to follow the standards defined by WOAH in Chapter 8.8 of the Terrestrial Animal Health Code [5]. To obtain the FMD-free status, with or without vaccination, absence of virus needs to be demonstrated according to predefined criteria [5]. In addition, an extensive risk analysis needs to be conducted, showing that associated risks connected to trade and regional context, among others, have been addressed and are properly managed [5].

The joint FAO/WOAH Global FMD Control Strategy offers the framework and tools to support FMD-control initiatives [6]. One of the core tools of this strategy is the Progressive Control Pathway for Food and Mouth (PCP-FMD), a progressive approach with several stages toward a FMD-free status [7]. The PCP-FMD is a framework based on risk and evidence, developed to assist FMD-endemic nations in gradually improving the management of FMD risks, reducing the impacts of the disease, and controlling virus circulation [7]. As nations progress through the framework, FMD risks are mitigated to the point where the submission of an application for an official recognition of freedom from FMD by the WOAH may be successful and sustainable [7]. In the current design of the PCP-FMD, freedom from FMD with vaccination is achieved at stage 4. After this, FMD freedom without vaccination is the next step to be considered [7]. Countries have been progressing through the PCP-FMD for many years, achieving freedom from FMD virus circulation. Today, several countries are FMD-free with vaccination or FMD-free without vaccination [2]. 

The decision to keep the FMD-free status with vaccination or move toward a FMD-free without vaccination status depends on several factors, and each country must decide for itself whether to continue or abandon vaccination. This decision needs to be made based on the specific situation and a detailed analysis (risks, cost–benefit ratio, etc.) that takes all aspects into account. Based on their assessments, countries may conclude that maintaining FMD-free with vaccination is the better option for their situation. Consequently, the status “FMD-free with vaccination” can also be regarded as “FMD-free with active prevention”, which might even be more appropriate. There is no virus circulation in either FMD-free status. The difference between the two FMD-free statuses is that the status “FMD-free with vaccination” includes active prevention through vaccination.

In recent years, significant advancements in vaccines, diagnostics, and disease control strategies have been achieved. That, in addition to the social and economic impacts of FMD outbreaks, makes it necessary to rethink the current approach that distinguishes between the two FMD-free statuses. Both FMD-free statuses require demonstration that no virus is circulating in a country or zone. It is time to treat them as equivalent. This paper presents the perspective that member countries and WOAH should recognize the two FMD-free statuses as equivalent. In the following sections, the scientific and technical bases supporting the equivalence of the two FMD-free statuses are outlined. 

## 2. How Has Freedom from FMD Been Achieved?

The importance of controlling FMD is recognized by most countries. National control programs are implemented for eventually eliminating the circulation of the virus. This process is supported by the Global Framework for the Progressive Control of Transboundary Animal Diseases (GF-TADs) [7] and other organizations.

FMD control is a long-term process, but many countries across the world have successfully and sustainably controlled or even eliminated the viral infection, as demonstrated by the low occurrence of outbreaks [8]. Most successful FMD-control strategies have relied on vaccines as control tools [9]. 

In Europe, extensive national FMD-control campaigns from 1960 to 1992 focused on cattle, relying on the use of high-quality vaccines to eliminate the virus. Thanks to their successful control programs, Europe decided to move toward a non-vaccination policy in 1992. Only a few single outbreaks have occurred since then, and all of them were controlled, rapidly eliminating the virus from circulation [10]. 

In 1988, South America established the Hemispheric Program for the Eradication of Foot-and-Mouth Disease [11], which has been effective in coordinating national “Vaccinate-to-live” campaigns, with more than 94.7% of South American territory being officially recognized as FMD-free by the end of 2019 and the clearly defined aim to completely eradicate FMD from the Americas by 2025 [12]. 

## 3. Improved Vaccines and Tools as Key to Controlling FMD

Early FMD vaccines were of low or even unsatisfactory quality mainly due to limitations in quality control, which resulted in low vaccination reliability. This also might have led to low vaccination coverage, because the stakeholders did not have confidence in the vaccine available at the time. Often, the vaccines of the past were not sufficiently purified for nonstructural proteins (NSPs), which impeded the differentiation between infected and vaccinated animals (DIVA). 

Currently, high-quality vaccines, proven to be highly effective, are available. Technological improvements have included several steps along the manufacturing process, such as the use of first-order inactivants [13], more effective adjuvants [14], and enhanced concentration and purification processes [15,16] applied by many manufacturers. Since the 1990s, important steps have been made regarding vaccine quality, with the standardization of quality assessment methods and greater regulatory demands imposed on vaccine manufacturers. The vaccines chosen for FMD control must meet the quality standards defined by the WOAH Terrestrial Manual [17]. In 2023, the European Commission for the Control of Foot-and-Mouth Disease (EuFMD) and the Food and Agriculture Organization of the United Nations (FAO) created a list of prequalified FMD vaccines that have been independently evaluated and demonstrated compliance with the standards established by the WOAH [18].

These updated high-quality vaccines, applied within appropriate vaccination programs, can be used to effectively reduce the viral reproduction number in cattle, meaning reducing the expected number of cases directly generated by one infected animal in a susceptible population [19], which decreases the risk of viral transmission within and between herds [9]. 

A clear connection between the evolution of vaccine technologies and changes in the FMD-control strategies could be observed over the years [10]. In regions where control measures are being implemented, repeated prophylactic vaccination prevents the occurrence of larger-scale disease outbreaks [20] and significantly reduces virus circulation, ultimately leading to control/elimination of the virus from the region [20,21]. Nevertheless, the success of achieving protective immunity through vaccination depends on the potency and quality of the chosen vaccine, among other aspects [8]. In addition, improvements have also been achieved in the purification of vaccines from nonstructural proteins (NSPs) [16], which guarantees that the vaccines do not interfere with the DIVA test systems [22,23]. 

Diagnostic tests based on DIVA technology, capable of detecting antibodies directed against the NSPs of the virus, have been developed and validated according to WOAH standards [24,25,26,27,28]. Commercial FMD diagnostic kits are widely available [29,30,31], also in portable format for use in the field [32]. The performances of the different available diagnostic tests are verified by the WOAH reference laboratories [33,34]. The application of NSP tests for FMD is well established [23], and surveillance strategies based on this technology are encompassed within the WOAH Terrestrial Animal Health code [5]. 

## 4. Disease Control

To monitor the occurrence and prevalence of FMD, the establishment of a warning system, an early disease detection and surveillance strategy, in accordance with the WOAH guidelines, is crucial [5]. This includes continuous clinical and serological surveillance. Due to this combination, an under detection of infection is very unlikely. An incursion would be detected efficiently, as shown in Argentina, as an example [35].

Despite the ability of the FMDV to establish persistent infection, for a limited period, the epidemiological relevance of persistently infected animals has been ruled out [36]. An exception is the presence of reservoirs, such as with the African buffalo [37], which can lead to a spillover from time to time [1]. However, in a vaccinated population the risk for spillover is even lower, and spread of the disease is limited [9]. 

The risk of disease re-emergence in a FMD-free area is always present, as long as the virus has not been completely eradicated. Therefore, there is always a risk of incursion for countries that are FMD-free with or without vaccination [38]. This risk needs to be managed appropriately through efficient control measures. It must be kept in mind that the impact of an incursion is much more severe in a naïve population, and the virus will spread faster and further there than in a vaccinated population [9]. 

In the past, between 1998 and 2000, the emergent FMD virus serotype O Pan Asia lineage triggered an explosive pandemic in Asia that spread to Africa and Europe, where some of the countries affected had been free from FMD for several decades [39]. The re-emergence risk is especially relevant in areas that share borders with FMD-infected countries or zones. Therefore, FMD-free statuses are dynamic, being subject to change based on FMD incursions [38]. 

There is evidence of the circulation of FMD virus strains beyond their traditional endemic pools [40]; as recent examples, the identification of the serotype A lineage Euro-South America in Egypt [41] and the SAT-2 serotype in Iraq [42], Jordan [43], and Türkiye [44] can be mentioned. These events highlight the importance of assessing the risk of a FMD incursion, taking the regional epidemiological situation into account [45], keeping active surveillance systems and being prepared in case of a FMD outbreak, as long as FMD is not globally eradicated.

As countries and zones progress through the PCP-FMD, vaccination is used to reduce the incidence of outbreaks, eventually eliminating the virus. The proportion of protected animals in a population depends on vaccination coverage and is based on an appropriate vaccination strategy [8]. To evaluate the protective immunity conferred by FMD vaccines, serological tests detecting antibodies directed against the virus’s structural proteins (SPs) can be performed [5]. 

## 5. The Need for a More Sustainable Approach

Controlling FMD outbreaks by stamping out large numbers of both infected and uninfected animals, as was conducted previously in several countries, is a multilevel conflict, not acceptable from food security, economic, and animal welfare standpoints [46]. Given that vaccination for FMD is effective and readily available, stamping out animals is no longer acceptable. Massive stamping out interventions would likely no longer be accepted by the general public and could have a huge negative impact on the public perception of livestock production [46].

In the case of a FMD outbreak, an individual farmer may have to deal with the loss of their entire herd; an event as such that can have tremendous economic and psychological repercussions [47], which overall could have an enormous impact on the sector. 

The economic impact of a FMD outbreak can be tremendous, as previous outbreaks have shown. The costs of a FMD outbreak are the costs directly connected to the outbreak (direct costs) and indirect costs affecting other industries, including tourism [48]. Adjusted to current inflation using previously defined methodologies [49], the outbreak that occurred in the United Kingdom in 2001 would cost around USD 15 billion today [50]. In the Republic of Korea, five FMD outbreaks occurred between 2000 and 2011, with the associated direct costs ranging from USD 32 million to USD 2.5 billion, per outbreak [51]. The Korean government adjudicated USD 231 million for quarantine, decontamination, and support for farmers, while registering USD 481 million in losses associated with the culling of more than 325 thousand animals [52].

The loss of FMD-freedom statuses will lead to economic losses due to imposed trade restrictions and will have an impact on the market price for meat and other animal products [53]. The direct economic impact of hypothetical FMD outbreak scenarios has previously been assessed in Brazil, with estimations ranging between USD 132 and 271 million [54], and in Australia where it was estimated that it could reach USD 53 billion [55,56]. These estimations were based on country-specific contexts and data. However, they can give an indication of the possible costs of an outbreak in any other FMD-free country or zone. 

Before deciding to stop vaccination, a country needs to assess the associated risks and potential economic consequences. Assuming a hypothetical outbreak with direct costs of USD 3000 million, in a country that produces 1 million tons of beef per year, the country would need to sell their meat at an extra cost of 0.6 USD/kg for 5 years, to compensate only for these direct economic costs. These figures show that the perceived added value of trading under a FMD-free without vaccination status probably does not justify the risk of stopping vaccination, and this estimation does not include indirect costs of the outbreak and reputation damage. 

Perry et al. [57] analyzed the potential commercial benefits for Uruguay of achieving a FMD-free status without vaccination, making the transition from the current FMD-free with vaccination status [57]. This change would involve the development and implementation of a control strategy based on active surveillance and biosecurity. In addition, this would require parallel investments in strengthening veterinary services to guarantee the operation of a surveillance system and overall strengthening of the animal health system. For a country with limited public resources, such as Uruguay, the achievement of these conditions would represent a great challenge, especially considering the very limited increased trade profits connected with achieving a free without vaccination status. In addition, ceasing vaccination would increase the risk of introduction, exposure, and dissemination of FMD throughout the country, increasing the vulnerability of the livestock sector to the consequences of a large-scale outbreak [57].

Conducting a cost–benefit analysis for a FMD vaccination control intervention, under different possible scenarios, may be worthwhile for countries. These analyses have been carried out all over the world with varying conclusions [48]. As an example, in Scotland such an analysis has been conducted, where it was estimated that controlling a FMD outbreak with emergency vaccination would have significantly lower direct and indirect costs compared to a stamping out response [58]. 

Investing in the development and application of vaccines to control animal disease outbreaks is a world-wide tendency, which is currently being considered to contain diseases that have been classically controlled mainly by culling susceptible animals, such as with avian influenza [59].

## 6. Demonstrating and Relying on Freedom from FMD

To achieve freedom from FMD, the absence of virus circulation needs to be proven according to internationally accepted criteria. This must be shown for both FMD-free statuses, with and without vaccination [5]. Showing absence of virus circulation is mainly conducted through regular surveillance, including serological testing and clinical observations [60]. For countries or zones that are free from FMD with vaccination, DIVA testing systems allow them to show freedom from virus circulation [5]. Sentinel animals may additionally serve as indicators of virus circulation [5].

International regulations on the trade of meat from susceptible animals with bone and giblets from a FMD-free with vaccination country or zone are based on the recommendations of the WOAH Terrestrial Manual, Chapter 3.1.8 [17] and the Terrestrial Animal Health Code, Chapter 8.8 [5]. Currently, only the head, pharynx, tongue, and associated lymph nodes are penalized in the event of trading meat from a country or zone that is FMD-free with vaccination to a FMD-free without vaccination country or zone. Bursa et al., 2023, conducted a quantitative risk assessment, where the probability of FMDV introduction into a FMD-free without vaccination country from a FMD-free with vaccination country (Argentina) via imported bone-in beef and offal trade, as currently penalized by WOAH’s recommendations, proved to be negligible [35].

Over the last thirty years, meat and meat products from vaccinated bovines have been traded safely from South America to other countries or zones that are FMD-free without vaccination, with more than 8.5 million tons of meat exported over the last ten years alone, without ever showing evidence of virus transmission [35,61,62]. Even the export of bovine tongue, as with the example from Uruguay (FMD-free with vaccination) to Japan (FMD-free without vaccination), shows that it can be safely carried out [63]. These figures imply that trade between FMD-free areas with vaccination is already recognized as safe by the international market.

## 7. FMD-Control Challenges

Achieving and maintaining FMD control through vaccination does not come without challenges. Of course, different production systems require different approaches to manage and maintain freedom, with and without vaccination, from virus circulation. A special focus needs to be put on the potential risk of new viral serotypes being introduced into a region [40]. This could trigger the need for an adaptation of the vaccine strains used for vaccination [64], although high-potency vaccines and/or revaccination schemes can compensate for a poor match between the field and vaccine strains [65]. The composition of susceptible animal populations and the production system specificities are of importance and should be properly valued in the design of the vaccination and surveillance strategy to demonstrate FMD freedom [8]. National and local FMD-control policies should be defined by species-specific data and adapted to the local animal population and production structures [66]. In particular, FMD in sheep is critical given that the clinical signs in this species are very discrete [67], and historically these animals have often not been included in vaccination programs [9]. However, given the logistical and economic constraints that may hinder complete vaccination coverage of a susceptible population, targeting a particular livestock sector such as dairy cattle or pigs may be considered and is a common practice [8].

Although a low proportion of vaccinated and infected cattle can remain asymptomatically infected during an indeterminate period [68], there is no evidence of viral transmission originating from these animals [36]. Also, there is evidence that the use of potent vaccines will reduce the likelihood of prolonged virus circulation in the animals [9]. 

## 8. The Prerequisites for Equivalence between the FMD-Free Statuses

Vaccines are important tools, among other tools used to control FMD and eventually achieve freedom from virus circulation. Regardless of vaccination status, to achieve an official FMD-free sanitary status, freedom from virus circulation always needs to be proven with documented evidence of compliance with WOAH’s requirements [5]. To achieve this, national veterinary authorities need appropriate competency in epidemiology and technical knowledge. 

Nevertheless, it is essential to highlight that recognizing the two FMD-free statuses as equivalent requires the use of high-quality, purified, potent vaccines, for example, the ones on the list of prequalified FMD vaccines maintained by EuFMD and FAO [18]. Such vaccines, in combination with effective disease surveillance strategies and competent veterinary services, are essential components for successfully eliminating the FMD virus, preventing its reintroduction, and eventually achieving a FMD-free status. 

Vaccines that are not sufficiently purified for NSPs will not allow demonstration of the absence of NSP antibodies in vaccinated animals and therefore will not prove the absence of virus circulation. In addition, vaccines of insufficient quality will not be effective in preventing infection. A country that is not using high-quality DIVA vaccines will not be able to achieve freedom from FMD nor demonstrate it. 

## 9. Why the Two Statuses Should Be Made Equivalent

The risk of a FMD incursion will be present until the disease is eradicated globally, especially in a world that is so closely linked. The introduction of FMD into a region can occur through multiple pathways [1]. However, viral dispersion can be controlled by appropriate measures and granting protection via vaccination, as shown in the previous sections. Therefore, vaccines can function as insurance and can effectively contain viral dispersion, minimizing the potential consequences of a FMD outbreak. This not only provides more safety for the country that vaccinates but also for other countries in the region and globally, including those that may be free of FMD without vaccination.

Stopping prophylactic FMD vaccination programs in regions where the virus is still present is inevitably associated with an increased risk of widespread outbreaks. Therefore, after achieving freedom from disease with vaccination, ceasing active disease prevention through vaccination should only be implemented after a careful and comprehensive analysis of epidemiological challenges, such as the presence of the virus in the region and quantities of risk material traded, along with the social, geographic, economic, and political characteristics of the region. If this risk analysis shows that it is better for a country to not stop active FMD prevention through vaccination, e.g., due to a higher risk of FMD introduction or a cost–benefit analysis, this country should be able to do so without being penalized for the decision to maintain preventive measures. 

The FMD-free with vaccination sanitary status supports sustainable meat production in many cases. Given the current distinction between FMD-free statuses, countries in high-risk regions might be tempted to seek FMD-free status without vaccination, because they expect better trading conditions [5]. This is a false incentive that is associated with high risks and very high potential costs in the case of an outbreak, as seen in the past. So, incentivizing countries to take unnecessary risks by ceasing vaccination programs should not be a goal. It goes against all efforts toward animal health, food security, animal welfare, and sustainability. 

## 10. Conclusions

The concept of recognizing “FMD-free without vaccination” as the highest status in terms of sanitary protection assurance needs to be changed, and the two statuses must be recognized as equivalent in the PCP-FMD; in the WOAH Terrestrial Animal Health Code, Chapter 8.8; and the Terrestrial Manual, Chapter 3.1.8 [5,7,17]. 

The technological improvements in vaccines and diagnostics allow demonstration of freedom from virus circulation regardless of vaccination status. Therefore, the distinction between the two FMD-free statuses is outdated and no longer appropriate. 

Making the two FMD-free statuses equivalent allows countries to choose if they aim for freedom with vaccination (active prevention) or without vaccination, based on their risks and without compromising safety of the production system. Furthermore, recognizing the two FMD-free statuses as equivalent will help to eradicate FMD and support food security, economic development, and access to animal protein, especially in FMD-endemic regions. It might also motivate countries to start a rigorous vaccination program. Therefore, both FMD-free statuses need to be recognized as equivalent. FMD-free is FMD-free. 

## Data Availability

Not applicable.
